# Association Between the Triglycerides‐to‐High‐Density Lipoprotein‐Cholesterol (TG/HDL‐C) Ratio and Chronic Kidney Disease: A Systematic Review and Meta‐Analysis of Observational Studies

**DOI:** 10.1002/edm2.70161

**Published:** 2026-01-22

**Authors:** Mansour Bahardoust, Sheida Shokohyar, Ali Delpisheh, Meisam Haghmoradi, Azin Ghaffari

**Affiliations:** ^1^ Department of Epidemiology, School of Public Health & Safety Shahid Beheshti University of Medical Sciences Tehran Iran; ^2^ Breast Cancer Research Center Iran University of Medical Sciences Tehran Iran; ^3^ Mass General Hospital Institute of Health Professions Boston MA USA; ^4^ Urmia University of Medical Sciences Urmia Iran; ^5^ Rajaie Cardiovascular Medical and Research Center Iran University of Medical Sciences Tehran Iran

**Keywords:** chronic kidney disease, high density lipoprotein, systematic review, triglyceride, triglyceride to high‐density lipoprotein cholesterol ratio

## Abstract

**Background:**

The relationship between the triglycerides‐to‐high‐density lipoprotein cholesterol (TG/HDL‐C) ratio and the risk of developing chronic kidney disease (CKD) remains unclear, as CKD is a major health challenge worldwide. This study aimed to investigate the association between the TG/HDL‐C ratio and the risk of developing CKD.

**Methods:**

To find studies that examined the association of TG/HDL‐C ratio (without stratification restrictions for TG/HDL‐C) with CKD or renal disorders, PubMed, Embase, Scopus, Google Scholar, and Web of Science databases, as well as study references, were searched by two independent investigators with no time limit until July 31, 2025 by related MeSH Terms. Heterogeneity among studies was assessed using the Cochran's *Q* and *I* ^2^ tests. Meta‐regression was employed to manage the heterogeneity.

**Results:**

Eleven studies involving 376,697 participants were included. The pooled prevalence of CKD was 12% (95% CI: 7–16). Subgroup analysis showed a significant positive correlation between increasing TG/HDL‐C ratio and increasing CKD prevalence. The prevalence of CKD in quartiles 1, 2, 3, and 4 was 8%, 10%, 12% and 15%, respectively. A high TG/HDL‐C ratio (q4) versus a low one (q1) was significantly associated with increased risk of CKD (OR: 1.26, 95% CI: 1.13, 1.39, *I* ^2^: 73.8%, *p*: 0.001). Subgroup analysis showed that the increased risk of CKD in men and women with high versus low TG/HDL‐C ratio was 27% and 23%, respectively (*p* < 0.05).

**Conclusion:**

A high versus low TG/HDL‐C ratio was significantly associated with an increased risk of CKD. This association was stronger in men than in women. Despite its limitations, the TG/HDL‐C ratio can be used as a simple, reliable and accessible biomarker for assessing CKD risk.

## Graphical abstract

A high versus low TG/HDL‐c ratio may be associated with an increased risk of developing CKD. As a result, the TG/HDL‐c ratio can be used as a simple, reliable, and accessible biomarker for assessing CKD risk.

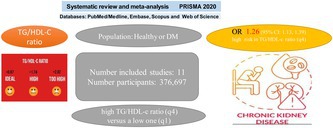

## 1 Introduction

Chronic kidney disease (CKD) is a progressive disease that is increasing worldwide with the aging population and the increasing incidence of non‐communicable diseases, including diabetes and hypertension. It is considered one of the major challenges for health systems [1, 2]. CKD is recognised as one of the major public health challenges and one of the leading causes of death in the 21st century [3, 4]. Its prevalence and incidence have increased by more than 40% over the last 30 years [4]. According to the latest studies, CKD affects the lives of more than 850 million people worldwide, with most cases reported in developing and low‐income countries [2, 5].

CKD is defined as any abnormality in kidney structure or function that persists for 3 months and is associated with clinical outcomes according to the KDIGO (Kidney Disease: Improving Global Outcomes) 2024 guidelines [6]. An estimated glomerular filtration rate (eGFR) of < 60 mL/min/1.73 m^2^ and an albumin‐to‐creatinine ratio (ACR) of 30 mg/g or greater are defined as the diagnostic thresholds for CKD [6, 7].

The best strategy for preventing and delaying advanced outcomes is to identify and manage risk factors for CKD [8]. Abnormalities in lipoprotein metabolism have been identified as a potential contributor to CKD, with moderate CKD associated with increased triglyceride (TG) levels and decreased high‐density lipoprotein cholesterol (HDL‐C) levels [9, 10, 11, 12, 13, 14]. The TG to HDL‐C (TG/HDL‐C) ratio has been identified as a simple, inexpensive and useful lipid parameter for predicting the risk of various diseases, including cardiovascular diseases, CKD and diabetes, in recent studies [15, 16, 17, 18, 19].

Several studies have shown that the TG/HDL‐C ratio can be significantly associated with the development of CKD and renal disorders [14, 15, 18, 20, 21]. However, the effect size of the association between the TG/HDL‐C ratio and the risk of CD has been heterogeneous across studies, and the association between the TG/HDL‐C ratio and CKD remains controversial [14, 15, 18, 20, 21]. Therefore, given the importance of this issue, this study aimed to investigate the association between the TG/HDL‐C ratio and the risk of CKD, using this lipid index.

## 2 Methods

Protocol Registration and Search for Potential Studies.

This systematic review was conducted after registration in PROSPERO (International Prospective Register of Systematic Reviews) based on the PRISMA (Preferred Reporting Items for Systematic Reviews and Meta‐Analyses) guidelines [22].

### 2.1 Literature Search

First, keywords were determined to design a search strategy based on the research question. A team of nephrologists, endocrinologists and epidemiologists validated the keywords.

To find studies that examined the association of TG/HDL‐C ratio with CKD or renal disorders, PubMed, Embase, Scopus, Google Scholar, and Web of Science databases, as well as study references, were searched by two independent investigators with no time limit until July 31, 2025.

The overall search strategy was determined using the PICO (Patient, Intervention, Comparison and Outcome) framework.

Patient: Healthy or diabetic participants, Intervention: High TG/HDL‐C ratio (Q4), Comparison: Low TG/HDL‐C ratio (Q1), Outcome: CKD prevalence, effect size of association of TG/HDL‐C ratio with CKD.

The search criteria included a combination of key terms: (‘triglycerides‐to‐high‐density lipoprotein‐cholesterol’ OR ‘triglyceride/high‐density lipoprotein cholesterol ratio’ OR ‘lipid ratios’ OR ‘TG/HDL‐C ratio’ OR ‘Dyslipidemia’) AND (‘Chronic Kidney Disease’ OR ‘CKD’ OR ‘Chronic Renal Insufficiencies’ OR ‘Chronic Kidney Insufficiencies’) AND (‘Biomarkers’ OR ‘Risk assessment’).

Participants in the original studies were stratified into four groups (quartiles 1–4) based on their TG/HDL‐C ratio. The prevalence of CKD in the different quartiles was estimated. The association of high versus low TG/HDL‐C ratio for the development of CKD was reported in the original studies as an odds ratio. In all studies, the TG/HDL‐C ratio was calculated using the same formula. The definition of CKD was also the same in all studies. It was based on (estimated glomerular filtration rate (eGFR) of < 60 mL/min/1.73 m^2^ (low eGFR) and/or proteinuria (defined as urinary protein ≥ 1+ on dipstick testing)).

### 2.2 Eligibility Criteria

Inclusion criteria included observational studies (cohort, case–control and cross‐sectional). These studies examined the association between the TG/HDL‐C ratio and CKD (without stratification restrictions for TG/HDL‐C). These studies reported adjusted effect sizes (odds ratios) for the association between TG/HDL‐C and CKD, published in English with no time restrictions until July 31, 2025. Studies with a population with underlying renal disease, studies with subgroups of fewer than 50 participants, studies with a population in the age range of < 20 years and more than 80 years, lack of access to full‐text articles, reviews and meta‐analyses, laboratory or animal studies, case reports and case series were defined as exclusion criteria.

### 2.3 Identifying Relevant Studies and Data Extraction

**FIGURE 1:**
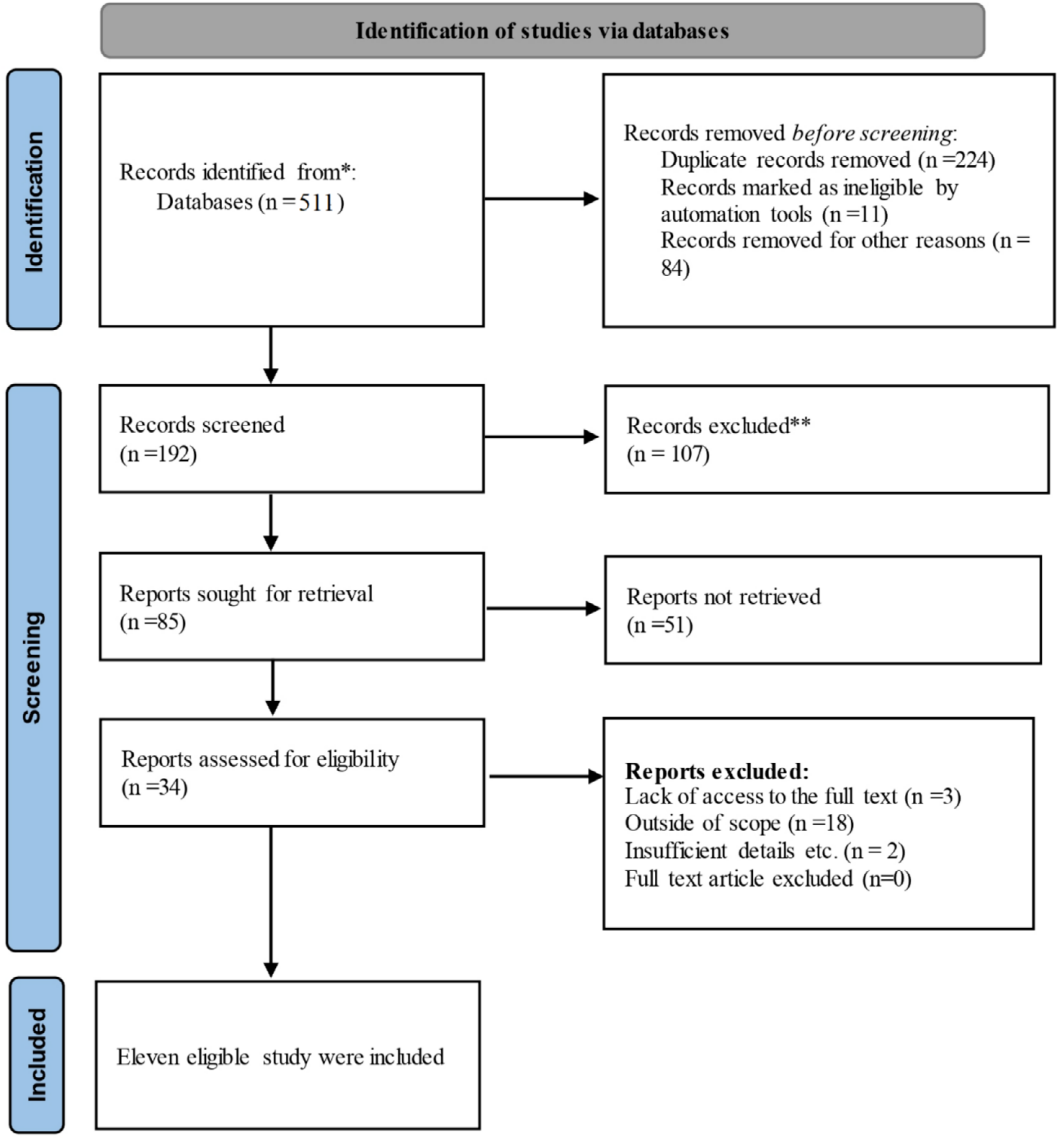
Flowchart page of studies based on PRISMA 2020.

After an initial database search, we identified 511 articles containing relevant keywords using the search strategy. Duplicate articles between sources were identified and removed using EndNote version 21. The remaining articles were screened by two independent researchers using the title and abstract. A third researcher resolved any disagreement between the two researchers regarding the inclusion of a study in the meta‐analysis. After applying the inclusion and exclusion criteria, articles were assessed based on their abstracts and research questions. Finally, the full text of 34 articles was reviewed. Eleven studies were included in this meta‐analysis (Figure 1).

The required variables were extracted step by step as follows: An Excel file containing the required variables identified in the literature review was created. Two independent researchers extracted the desired variables from the selected studies. A third researcher resolved any discrepancies between the two researchers for a variable. Required variables included study authors, study year, study site, study design, mean Age, gender distribution, smoking, mean BMI, total number of subjects studied, number of patients with CKD, TG/HDL‐C ratio classification, TG/HDL‐C quartiles, number of CKD in TG/HDL‐C quartiles, eGFR level, number of CKD by sex, effect size of high versus low TG/HDL‐C (odds ratio) at 95% confidence interval (95 5 CI), effect size of association by cut‐off of TG/HDL‐C and gender and definition of CKD. Where possible, we contacted study authors for missing data.

### 2.4 Quality Assessment

The ROBINS‐I (‘Risk Of Bias In Non‐randomised Studies ‐ of Interventions’) checklist was used to assess the quality of the observational studies included in this systematic review [23]. We also assessed certainty of evidence using the GRADE approach [24].

### 2.5 Statistical Analyses

Data were analysed using Stata version 17 with random‐effects models. The pooled prevalence of CKD and its prevalence in quartiles of TG/HDL‐C ratio and by gender were estimated using the MetaProp command, with 95% confidence intervals (95% CI). The effect size of the association between a high versus low TG/HDL‐C ratio and CKD was reported as odds ratios (ORs) with 95% CIs. Cochran's *Q* and *I* ^2^ tests were used to assess heterogeneity across studies. We interpreted *I* ^2^ values as follows: 0%–40% may indicate little heterogeneity; 40%–75% may indicate moderate heterogeneity; and > 75% may indicate substantial heterogeneity. Meta‐regression analysis was performed to identify and control for factors associated with heterogeneity. Publication bias was assessed with the Egger test, and the results were visually displayed in a funnel plot. Tables and figures were used for descriptive purposes. As no publication bias was observed across studies for any of the outcomes, there was no need to use the Trim and Fill analysis to address this issue. Sensitivity analysis was performed to account for the effect of individual studies on the Clay outcome. Subgroup analysis for effect size was performed based on two criteria: TG/HDL‐C ratio ≤ 2.5 and > and gender.

## 3 Results

Finally, eleven observational studies [15, 16, 18, 20, 21, 25, 26, 27, 28, 29, 30] including 376,697 participants included in this meta‐analysis. The mean age of the participants was 54.2 years. In terms of gender distribution, 168,781 (44.8%) were men, and the rest were women. The mean BMI was 25.4. The mean eGFR was 84.3. In terms of smoking, 106,131 (28.2%) were current smokers. In terms of risk of bias, the majority of studies were at low risk, and only two studies were at moderate risk. In terms of certainty of evidence, almost all studies were at high or moderate certainty. Demographic characteristics, risk of bias and certainty of evidence are reported separately in Table 1.

**TABLE 1:** Baseline characteristics, certainty of evidence, and risk of bias of studies included in this meta‐analysis.

Authors (Year)	Design	Country	Sample size	participants	NCKD	TG/HDL‐C (q1, 2, 3 and 4) cut off for comparison	Age	Sex (male/female)	BMI	DM	Smoking status	eGFR	quality	Certainty of evidence
HT Kang (2011) [25]	Cross sectional	South Korea	5503	Healthy or DM participants	497	(Q4: > 4.5 vs. Q1:< 1.38)	46.9	2345/3158	23.8	NA	2781	77.3	Good	High
G Zoppini (2012) [15]	Prospective cohort	Italy	979	DM patients	106	(Q4: > 1.14 vs. Q1:< 0.11)	65.2	597/382	28.3	979	NA	81.01	Good	Moderate
K Tsuruya (2014) [16]	Prospective cohort	Japan	216,007	Healthy	39,110	(Q4: > 3.18 vs. Q1:< 1.2)	63.8	88,516/127491	23.1	52,058	67,394	75.5	Good	High
L Zhang (2014) [18]	Prospective cohort	China	1834	Healthy or DM participants	235	(Q4 vs. Q1)	53.1	679/1155	24.2	115	223	NA	Moderate	Moderate
CI Ho (2015) [21]	Cross sectional	Taiwan	46,255	Healthy	2149	(Q4: > 2.76 vs. Q1:< 1.04)	39.1	25,936/20289	24.2	NA	8558	108.1	Good	High
J Wen (2018) [20]	Cross sectional	China	48,054	Healthy or DM participants	1799	(Q4: > 1.37 vs. Q1:< 0.47)	44.3	28,823/19231	NA	NA	15,762	82.4	Good	High
L Yu (2019) [28]	Cross sectional	USA	13,780	Healthy or DM participants	2176	(Q4: > 2.7 vs. Q1:< 1.4)	45.4	6480/7300	27.9	1519	2703	102.2	Good	High
VD Raikou (2020) [27]	Cross sectional	Greece	183	Healthy	31	(Q4 vs. Q1)	67.3	97/86	28	0	33	52.3	Moderate	Moderate
S Lv (2021) [26]	Prospective cohort	China	7316	Healthy or DM participants	756	(Q4: > 2.97 vs. Q1:< 1.6)	58.3	3315/4001	23.8	1176	2788	75.2	Good	High
Q Zhai (2022) [29]	Prospective cohort	China	3909	Healthy or DM participants	574	(Q4: > 3.4 vs. Q1:< 3.4)	55	1168/2741	25.1	NA	610	101.1	Good	High
X Zhang (2024) [30]	Cross sectional	China	32,877	Healthy or DM participants	4734	(Q4: > 1.65 vs. Q1:< 0.69)	57.6	10,825/22052	NA	7380	4898	94.1	Good	High

### 3.1 Prevalence of CKD

**FIGURE 2:**
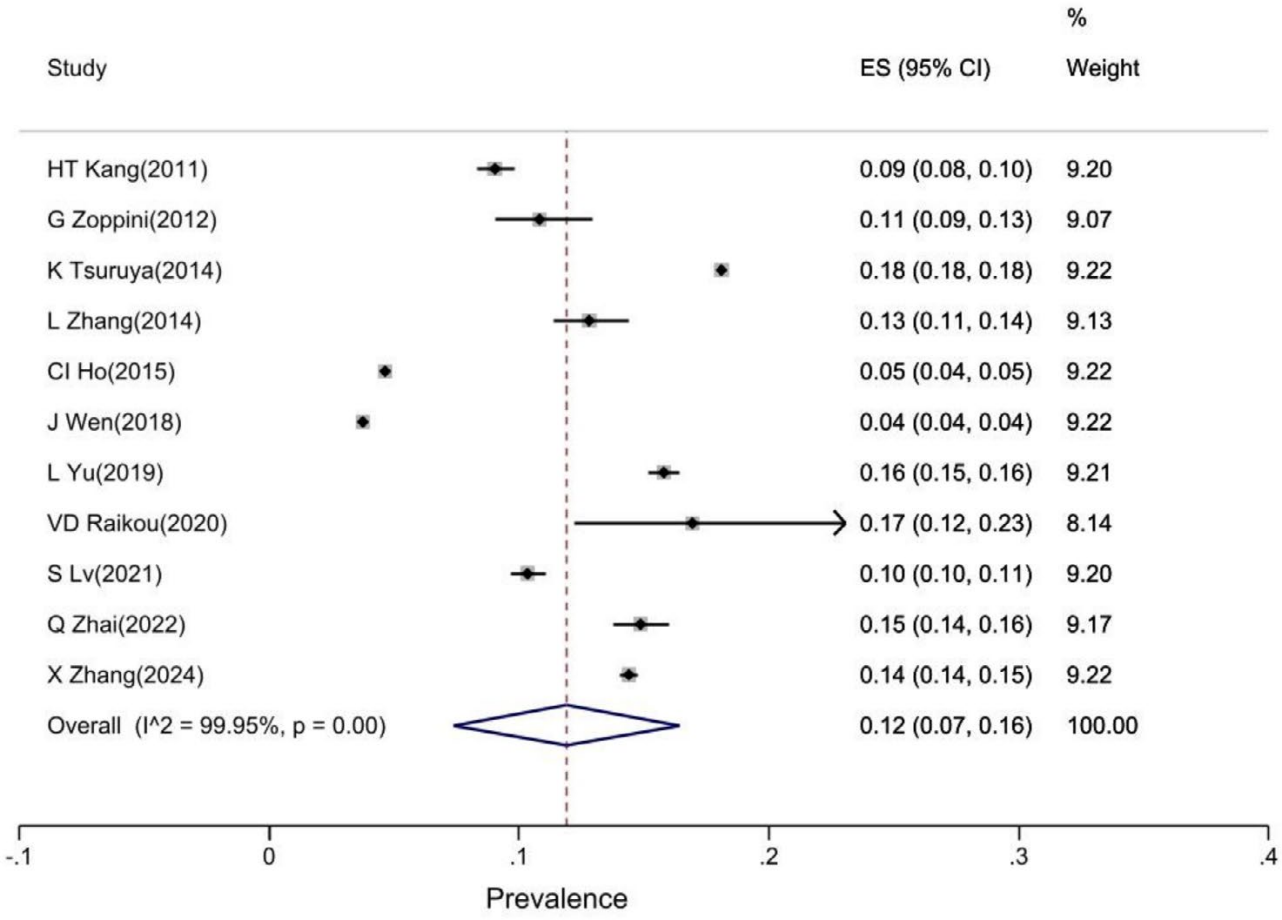
Forest plot of overall prevalence of CKD.

Based on a pooled estimate of 11 studies [15, 16, 18, 20, 21, 25, 26, 27, 28, 29, 30], the prevalence of CKD was estimated to be 12% (prevalence: 12% 95% CI: 7.16) (Figure 2).

Seven studies [16, 18, 20, 21, 25, 28, 30] examined the prevalence of CKD by gender. The results of subgroup analysis by gender showed that the prevalence of CKD was similar in both sexes and was 11% (Figure S1).

**FIGURE 3:**
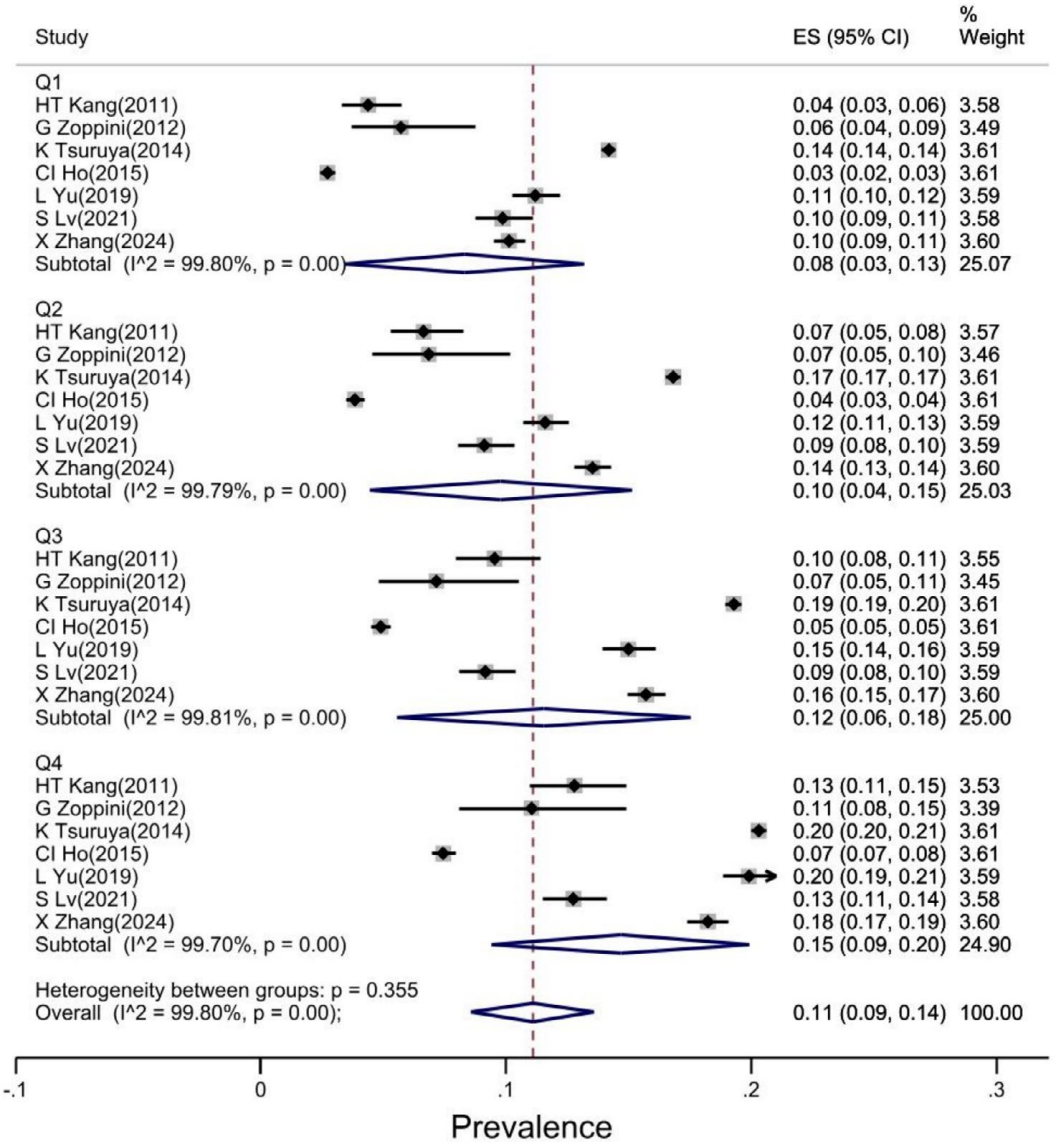
Forest plot of CKD prevalence based on TG/HDL‐C ratio quartile subgroups.

Subgroup analysis by TG/HDL‐C ratio quartile was performed in seven studies [15, 16, 21, 25, 26, 28, 30]. The pooled estimates showed that the prevalence of CKD in Q1, Q2, Q3, and Q4 was 8%, 10%, 12% and 15%, respectively (Figure 3).

### 3.2 Association of TG/HDL‐c Ratio With CKD

**FIGURE 4:**
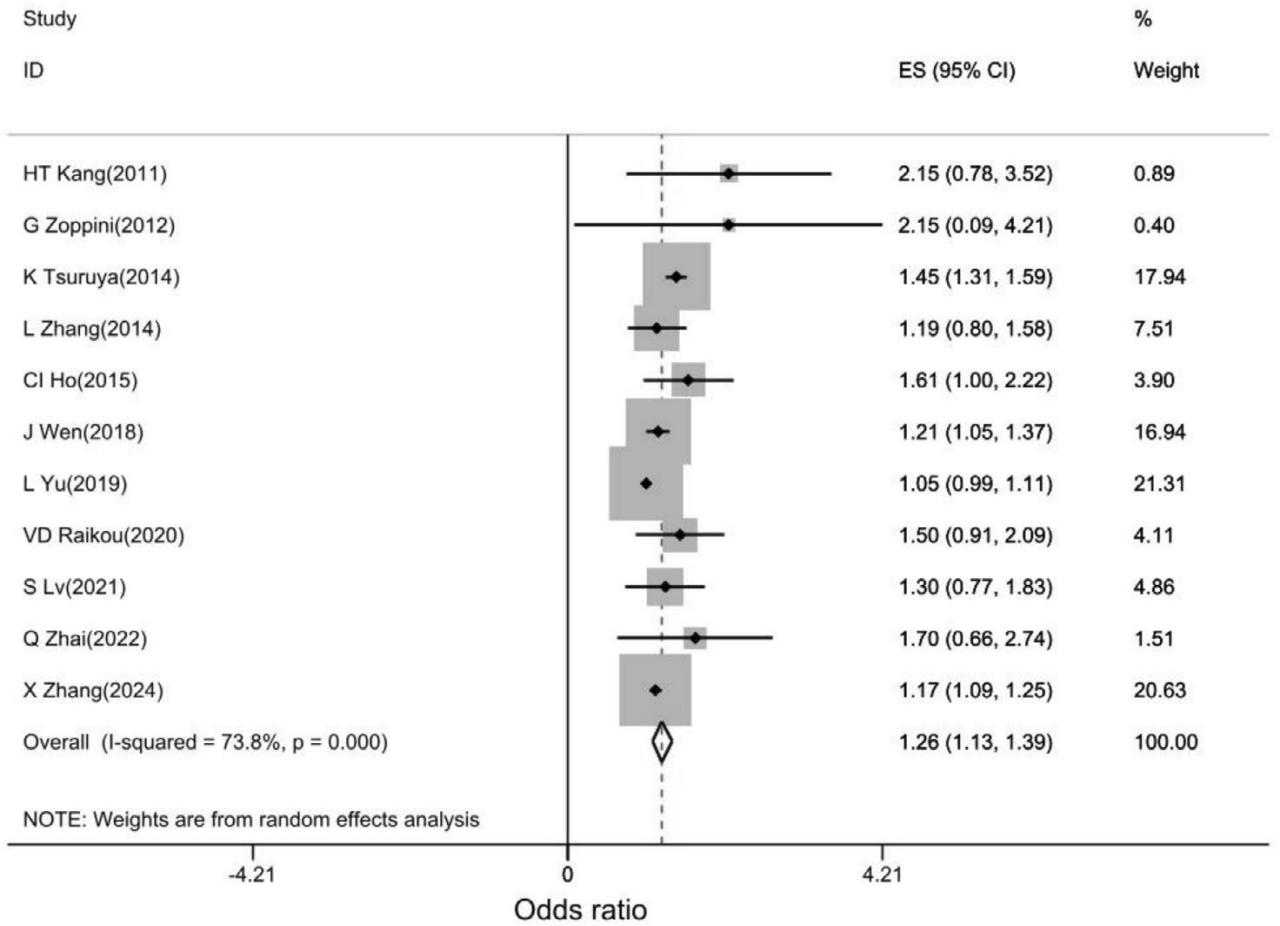
Forest plot of association of TG/HDL‐C ratio with CKD.

A pooled analysis of eleven studies showed that the high TG/HDL‐C ratio (Q4) was significantly associated with an increased risk of developing CKD compared to the low TG/HDL‐C ratio (Q1) (OR: 1.26, 95% CI: 1.13, 1.39, *I* ^2^: 73.8%, *p*: 0.001; Figure 4).

**FIGURE 5:**
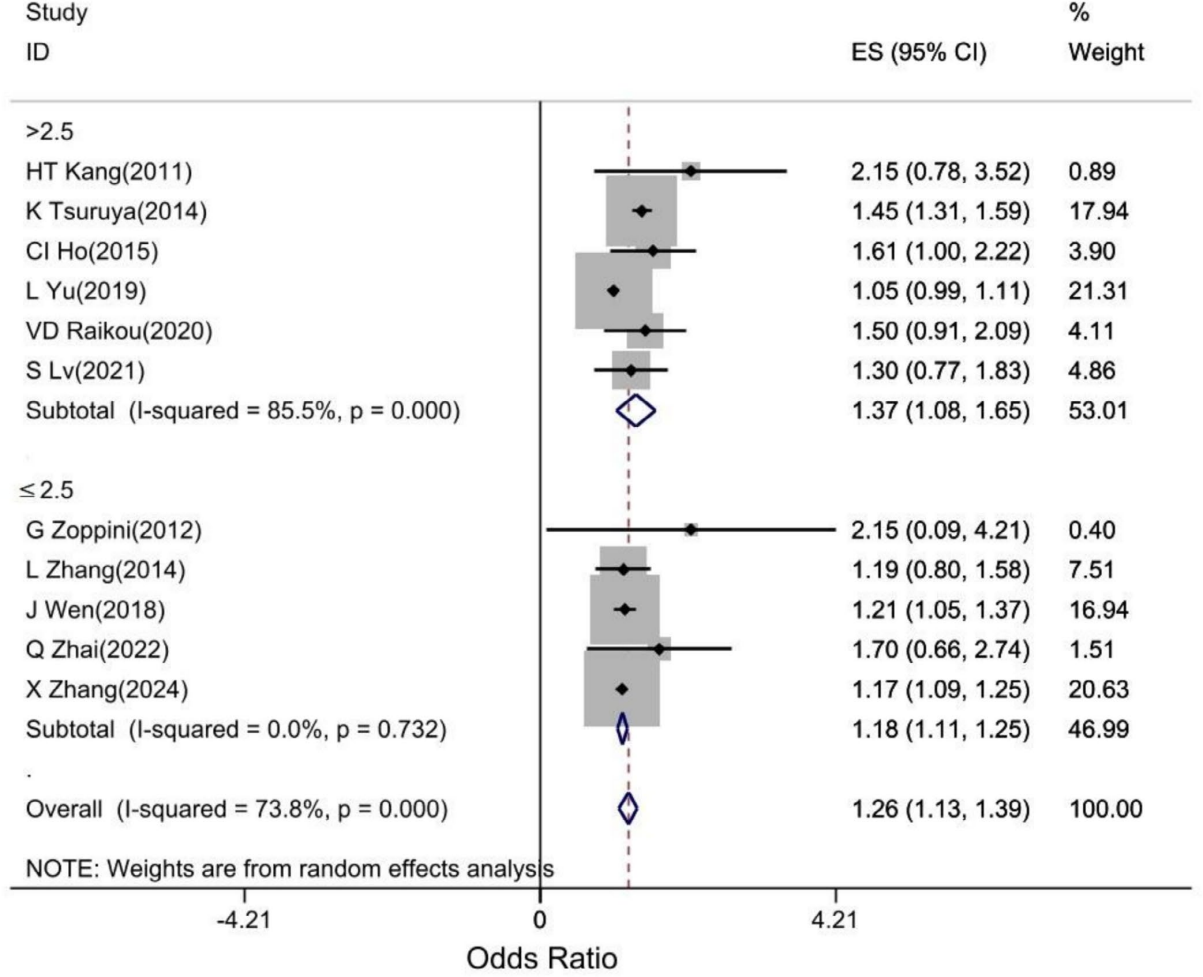
Forest plot of association of TG/HDL‐C ratio with CKD based on TG/HDL‐C ratio.

The results of the subgroup analysis based on the cut‐off of TG/HDL‐C ratio > 2.5 versus ≤ 2.5 showed that the chance of CKD was higher in individuals with TG/HDL‐C ratio > 2.5 than in individuals with a TG/HDL‐C ratio ≤ 2.5 (OR: 1.37, 95% CI: 1.08, 1.65) versus (OR: 1.18, 95% CI: 1.11, 1.25) Figure 5.

The association of TG/HDL‐C ratio with CKD in gender subgroups was examined in six studies [15, 16, 18, 21, 28, 30]. Pooled estimates of the results showed that the TG/HDL‐C ratio was associated with an increased risk of CKD in both men (OR: 1.27, 95% CI: 1.03, 1.15) and women (OR: 1.23, 95% CI: 1.07, 1.39) (Figure S2).

### 3.3 Sensitivity Analysis and Meta‐Regression

A sensitivity analysis was performed to estimate the effect size of each study on the overall estimate of the association between the TG/HDL‐C ratio and CKD (Figure S3).

Meta‐regression analysis aimed at identifying and controlling for factors that contributed to heterogeneity between studies and related to the overall effect size showed that study sample size, study quality, cut‐off for comparison of TG/HDL‐C ratio in each study, mean age, smoking, and gender were significantly associated with the effect size of the association of TG/HDL‐C ratio with CKD (*p* < 0.05; Table 2).

**TABLE 2:** Meta‐regression model of the effect of variables on effect size.

Variable	β	SE	*p*
Sample size	0.33	0.13	0.011
Mean age (per 10 year)	0.11	0.05	0.031
TG/HDL‐c ratio > 2.5	0.45	0.15	0.009
Current smoking	0.41	0.13	0.034
Study quality (High)	−0.21	0.14	0.045
Sex (Male)	0.28	0.2	0.046

### 3.4 Publication Bias

**FIGURE 6:**
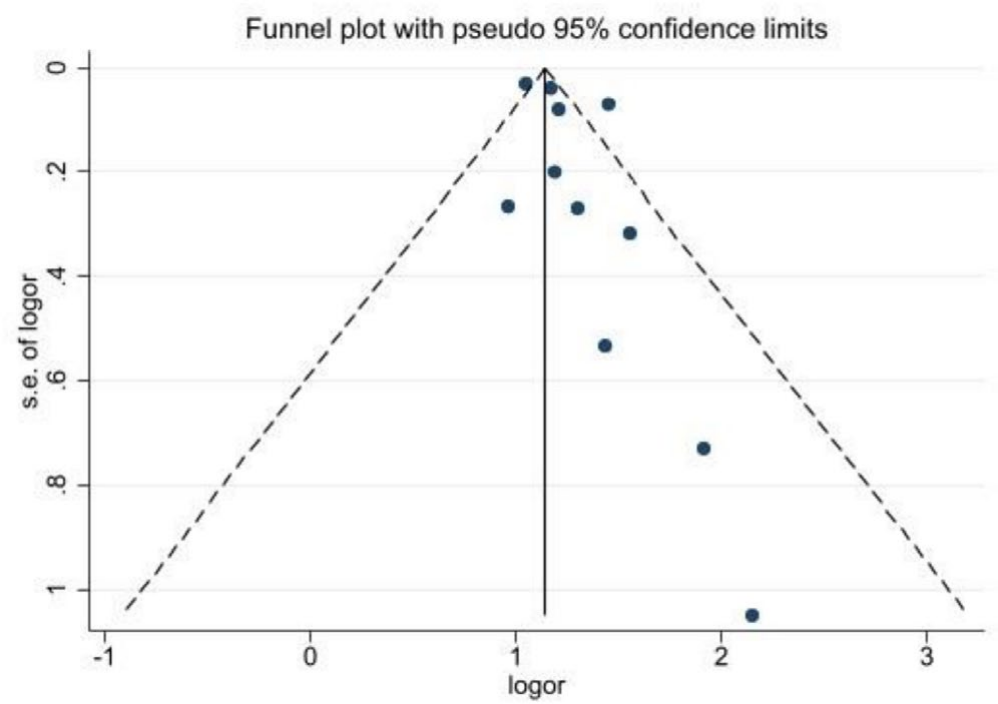
Bias publication assessment in the funnel plot.

Egger test results showed no significant effect of publication bias on the overall estimate of the association of TG/HDL‐C ratio with CKD (Egger test: 1.51, 95% CI: −0.15, 3.14, *p*: 0.055). The distribution of studies was also reported in a funnel plot (Figure 6).

## 4 Discussion

The effect size of the association between the TG/HDL‐C ratio as a composite indicator and CKD and renal impairment has varied in previous studies, possibly due to the small sample size of the studies examined, making this association still controversial. Given the importance of the topic, we examined the association of TG/HDL‐C ratio with CKD in 11 studies in this systematic review and meta‐analysis.

The pooled estimate of the results showed that the overall prevalence of CK was 12%. Subgroup analysis showed that the prevalence of CKD was significantly positively correlated with increasing TG/HDL‐C ratio. The prevalence of CKD in quartiles 1, 2, 3 and 4 was 8%, 10%, 12% and 15%, respectively. The prevalence of CKD was similar in both sexes, regardless of TG/HDL‐C ratio, although the confidence interval was narrower in women than in men.

Pooled estimates of the results showed that the chance of developing CKD was approximately 26% higher in individuals with a high TG/HDL‐C (Q4) ratio than in individuals with a low TG/HDL‐C ratio (Q1). Subgroup analysis results showed that although high TG/HDL‐C was associated with an increased chance of CKD in both sexes, the increased chance of CKD in men was approximately 4% higher than in women.

The majority of studies included in this meta‐analysis were of low risk of bias and high certainty of evidence. Although the heterogeneity between studies for the overall estimate was moderate (< 75%), we performed meta‐regression analysis to identify factors associated with heterogeneity. Meta‐regression analysis revealed that the heterogeneity between studies could be attributed to differences in subject characteristics (mean age, gender distribution, and percentage of smokers), study quality, and the TG/HDL‐C ratio cut‐off used in each study. However, several factors, including lifestyle, cultural differences, access to health services, and underlying diseases such as obesity and hypertension, may also contribute to this heterogeneity. Due to the lack of estimates in the original studies, we were unable to assess their impact.

In recent years, meta‐analyses have reported the association of the TG/HDL‐C ratio with several outcomes and diseases [17, 31]. In a systematic review and meta‐analysis, H Zhong et al. [17] evaluated the association between the TG/HDL‐C ratio and the risk of diabetes mellitus in 20 patients and showed that a high TG/HDL‐C ratio significantly increased the risk of developing type 2 diabetes compared to a low TG/HDL‐C ratio. The risk of developing type 2 diabetes was 1.78 times higher in patients with a high TG/HDL‐C ratio compared to a low TG/HDL‐C ratio. They also showed, similar to our results, that the increased risk of type 2 diabetes was greater in both sexes in individuals with a high TG/HDL‐C ratio compared to a low TG/HDL‐C ratio, and the magnitude of the risk was greater in men than in women, which may be justified due to differences in lifestyle and nutritional status between men and women [31, 32]. In a systematic review and meta‐analysis, Y Chen et al. [19] examined the association of the TG/HDL‐C ratio with cardiovascular events in the general population in 13 studies with a total of 207,515 participants. They showed that the risk of cardiovascular events was significantly (approximately 26%) higher in individuals with a higher TG/HDL‐C ratio compared with individuals with a lower TG/HDL‐C ratio, which confirmed the results of our study.

Recent studies have shown that the association of high TG/HDL‐C with an increased risk of CKD and impaired renal function can be explained by several mechanisms, including impaired lipoprotein metabolism, inflammation, oxidative stress and atherosclerosis [33, 34, 35, 36]. First, filtered proteins such as albumin and lipoproteins contain phospholipids and cholesterol, which can stimulate tubulointerstitial inflammation and damage the reabsorption process [33, 37]. Second, due to lipoprotein accumulation in the glomerular mesenchyme, mesangial cells are stimulated to produce matrix proteins, which leads to increased production of proinflammatory cytokines and the elimination of circulating macrophages and activation of resident macrophages. This process ultimately leads to glomerulosclerosis [38, 39, 40]. Third, previous research has shown a significant correlation between a high TG/HDL‐C ratio and elevated levels of small, dense LDL‐C particles, which are known to be highly atherogenic and a risk factor for CKD [41, 42, 43]. Fourth, the TG/HDL‐C ratio serves as a dependable measure of insulin resistance, which can lead to oxidative stress. Oxidative stress hinders the activation of nuclear factor‐erythroid‐related protein‐2, a protector against damage to kidney tissue [44, 45, 46, 47]. Fifth, dyslipidemia, particularly characterised by elevated TG and reduced HDL‐C levels, plays a role in atherosclerosis (the buildup of plaque in the arteries). Atherosclerosis within the renal arteries can diminish blood flow to the kidneys, resulting in kidney damage [48, 49, 50, 51].

### 4.1 Limitation

This systematic review had some limitations and strengths that should be noted. We were unable to adjust for several key variables, including nutritional status and lifestyle, that may have influenced the overall results due to the lack of reporting in the primary studies in this meta‐analysis. Also, our results were based on a pooled analysis of quartiles. Because the original studies reported only continuous quantitative variables, we were unable to conduct additional analyses based on these values. Most studies were conducted in developed countries with specific characteristics, particularly in East Asian countries, and caution should be exercised when generalising the results to other populations. Studies in other populations are recommended to more accurately estimate the results. Furthermore, the cut‐off for the TG/HDL‐C ratio classification varied across studies, although we ultimately arrived at a single classification. The most important strength of this study is that it assessed the association between the TG/HDL‐C ratio and CKD in a suitable sample size, using a systematic review and meta‐analysis with acceptable heterogeneity.

## 5 Conclusion

This systematic review and meta‐analysis showed that a high versus a low TG/HDL‐C ratio (Q4 vs. Q1) was significantly associated with an increased chance of developing CKD. This association was also present in subgroups of TG/HDL‐C ratio quartiles and gender. TG/HDL‐C ratio can be used as a simple, reliable and accessible biomarker for assessing the risk of developing CKD.

## Author Contributions

All authors contributed to the study conception and design. M.B., A.D.: conceptualization, methodology, software, writing – original draft, data curation, visualisation were performed; A.G., A.D., S.S., M.H. and M.B.: investigation, writing – original draft, writing – reviewing and editing were performed; A.G.: conceptualization, supervision, project administration were performed. All authors read and approved the final manuscript.

## Funding

The authors have nothing to report.

## Ethics Statement

Protocol registered in PROSPERO.

## Conflicts of Interest

The authors declare no conflicts of interest.

## Supporting information


**Data S1:** edm270161‐sup‐0001‐Figures.docx.

## Data Availability

The data that support the findings of this study are available on request from the corresponding author. The data are not publicly available due to privacy or ethical restrictions.
