# Supplementary material for: Association Between the Triglycerides‐to‐High‐Density Lipoprotein‐Cholesterol (TG/HDL‐C) Ratio and Chronic Kidney Disease: A Systematic Review and Meta‐Analysis of Observational Studies
Source: Endocrinol Diabetes Metab. 2026 Jan 22;9(1):e70161. doi: 10.1002/edm2.70161 (PMC12828072; doi:10.1002/edm2.70161)
Supplement: Supplementary file 1 — Data S1: edm270161‐sup‐0001‐Figures.docx. [file EDM2-9-e70161-s001.docx]

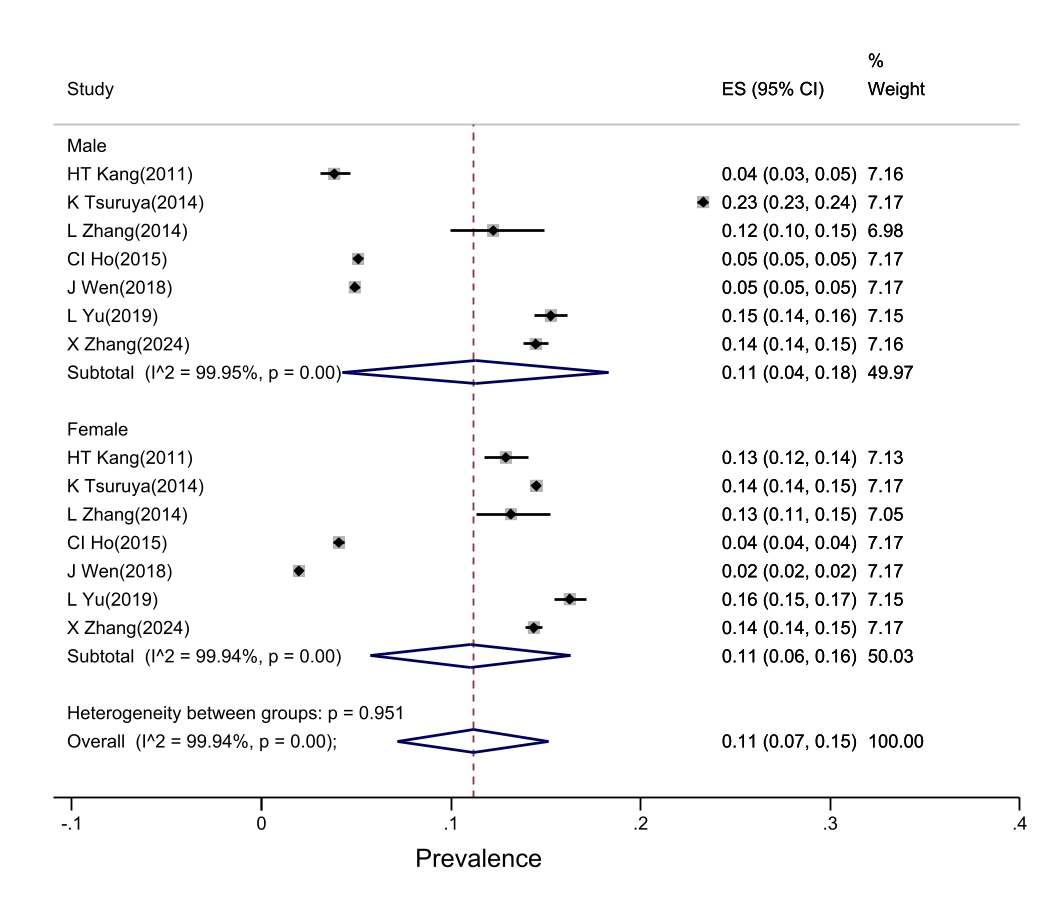


Figure 1: Forest plot of CKD prevalence based on gender subgroups


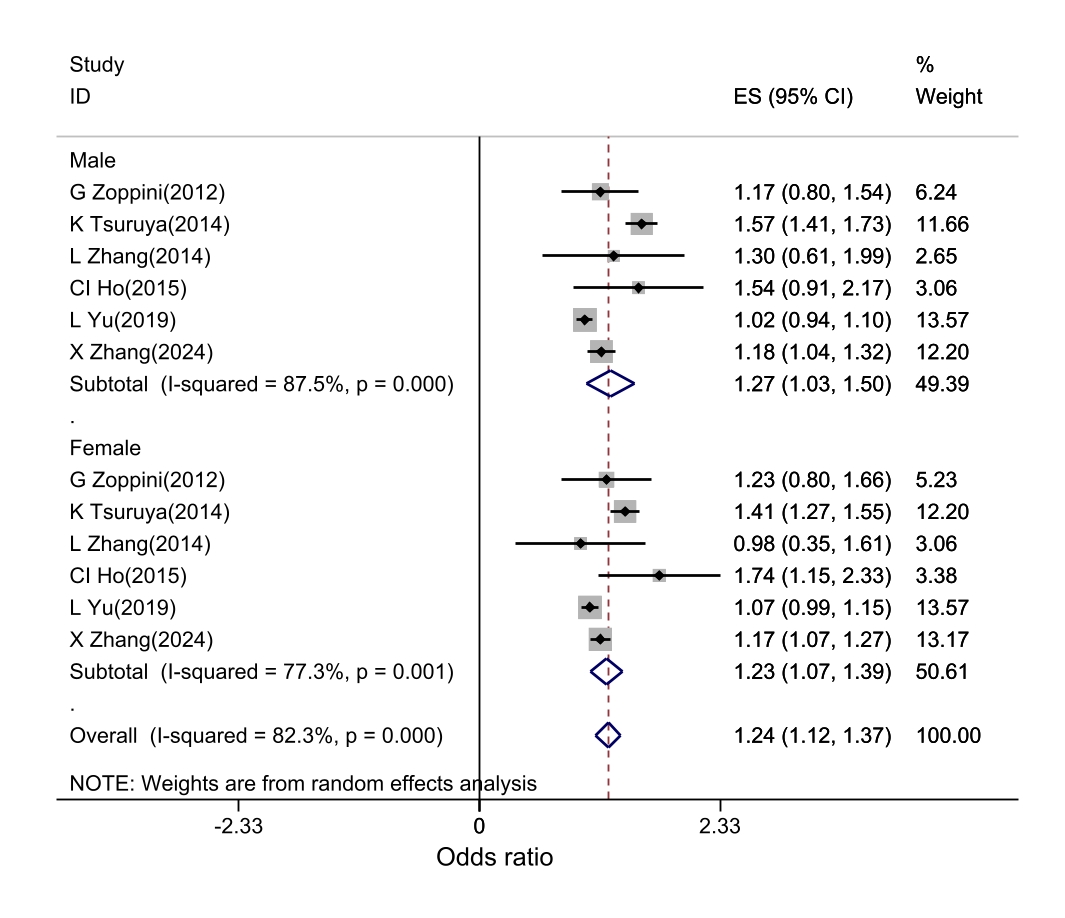


Figure 2::Forest plot of association of TG/HDL-c ratio quartile with CKD based on gender


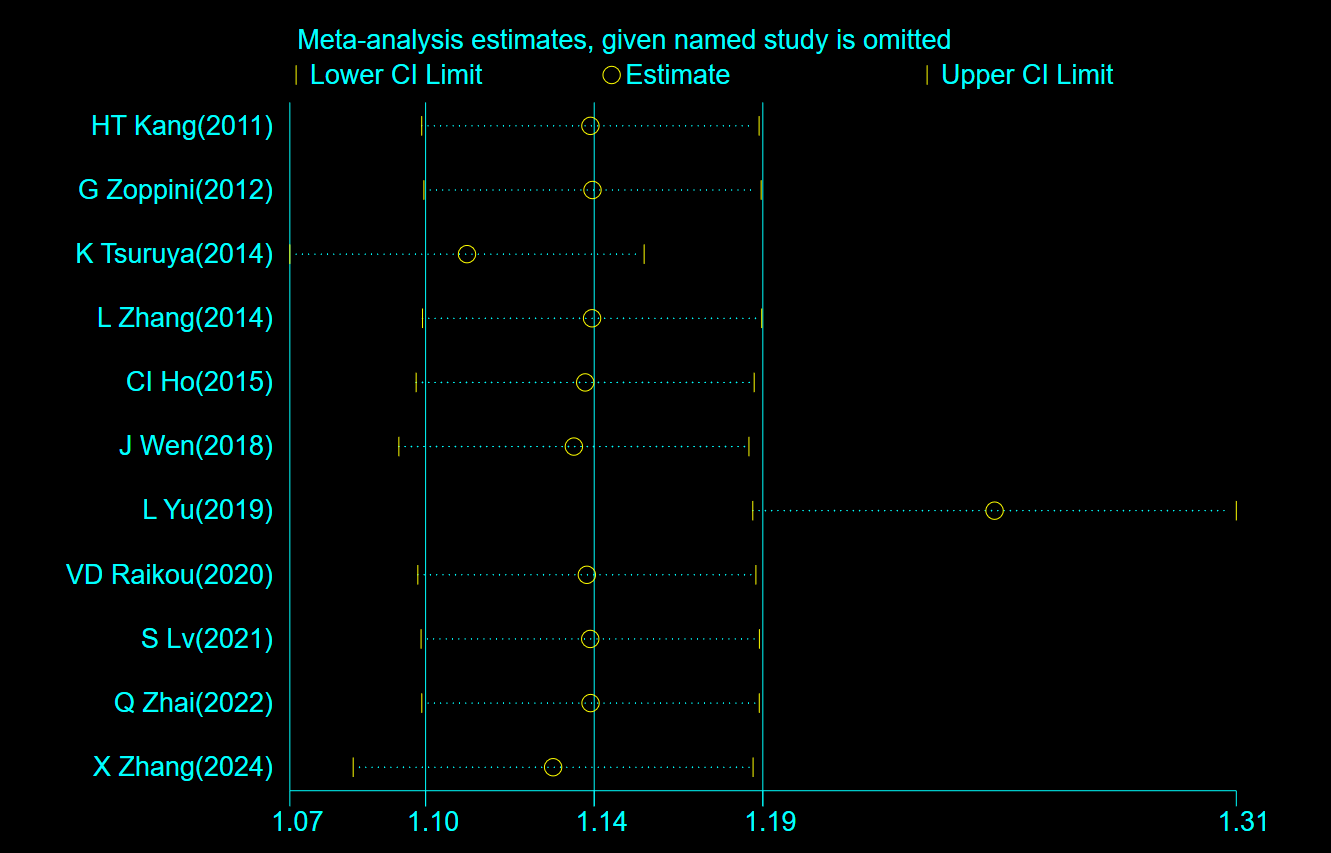


Figure 3: Results of sensitivity analysis to estimate the effect of individual studies on the overall effect size outcome
